# Trends in Early-Stage Cervical Cancer Management in the US: A National Cancer Database Analysis

**DOI:** 10.3390/curroncol31050215

**Published:** 2024-05-16

**Authors:** Jennifer Wolf, Yiyuan Wu, Judy Hayek, Qi Zhang, Ioannis Alagkiozidis

**Affiliations:** 1 Division of Gynecologic Oncology, State University of New York (SUNY) Downstate Health Sciences University, Brooklyn, NY 11203, USA; 2 Department of Population Health Sciences, Weill Cornell Medical Center, New York, NY 10065, USA; 3 Department of Obstetrics and Gynecology, Maimonides Medical Center, Brooklyn, NY 11219, USA; 4 Division of Gynecologic Oncology, Maimonides Medical Center, Brooklyn, NY 11219, USA

**Keywords:** cervical cancer, minimally invasive surgery, adjuvant radiation, LACC, postoperative outcomes

## Abstract

The Laparoscopic Approach to Cervical Cancer (LACC) trial was published in 2018 and demonstrated that minimally invasive surgery (MIS) yields inferior survival outcomes in early-stage cervical cancer compared to open surgery. This study investigates how the results of the LACC trial have impacted the selection of the primary treatment modality and adjuvant radiation utilization in early-stage cervical cancer. Using the National Cancer Database (NCDB), we compared patients with stage IA2-IB1 cervical cancer before (1/2016–12/2017) and after (1/2019–12/2020) the LACC trial. A total of 7930 patients were included: 4609 before and 3321 after the LACC trial. There was a decline in MIS usage from 67% pre-LACC to 35% thereafter (*p* < 0.001). In both the pre- and post-LACC periods, patients undergoing radical MIS more frequently had small volume disease (pre-LACC tumors ≤ 2 cm, 48% MIS vs. 41% open, *p* = 0.023; post-LACC stage IA2, 22% vs. 15%, *p* = 0.002). Pre-LACC, MIS radical hysterectomy was associated with White race (82% vs. 77%, *p* = 0.001) and private insurance (63% vs. 54%, *p* = 0.004), while there was no difference in socioeconomic factors in the post-LACC period. Although the proportion of patients treated with primary chemoradiation remained stable, the post-LACC cohort had a younger median age (52.47 vs. 56.37, *p* = 0.005) and more microscopic disease cases (13% vs. 5.4%, *p* = 0.002). There was no difference in the rate of radiation after radical hysterectomy before and after the trial (26% vs. 24%, *p* = 0.3). Conclusions: Post-LACC, patients were less likely to undergo MIS but received adjuvant radiation at similar rates, and primary chemoradiation patients were younger and more likely to have microscopic disease.

## 1. Introduction

When cervical cancer is diagnosed at an early stage, overall survival can be excellent, exceeding 90%. Surgical management in the form of radical hysterectomy is the preferred modality for early-stage cervical cancer, defined as stage IA1-IIA. Over the past two decades, there has been increasing focus on the use minimally invasive surgery (MIS) due to improved peri-operative outcomes, such as decreased length of hospital stay and blood loss. However, the recent literature has more closely defined the role of MIS in gynecologic cancers, including cervical cancer.

The Laparoscopic Approach to Cervical Cancer (LACC) trial was an impactful prospective study on early-stage cervical cancer. The results were first presented and published in 2018, demonstrating a decreased disease-free survival and overall survival for patients with newly diagnosed FIGO stage IA1 (with lymphovascular invasion), IA2, and IB1 disease treated with minimally invasive compared to open surgery [1]. Since the initial presentation and publication, much debate has commenced on the rationale for worse outcomes with minimally invasive surgery, with a focus on protective measures during colpotomy creation, use of a uterine manipulator, degree of surgical expertise, and previous conization biopsy [2,3,4,5]. Despite efforts to identify a subgroup that may still benefit from minimally invasive surgery, the LACC trial remains the only prospective randomized trial for the surgical management of this population of early cervical cancer, and many Gynecologic Oncologists have changed their patient counseling and practice patterns. A retrospective study using the National Inpatient Sample comparing the three years before the presentation of the LACC trial to the nine months following data release demonstrated a sharp decline in the use of minimally invasive surgery in this population [6]. Similarly, a multi-institutional retrospective study in Italy revealed a significant decrease in minimally invasive radical hysterectomies performed for patients with stage IA-IB2 cervical cancer (from 64.9% to 30.4%) compared to open radical hysterectomies, without a significant increase in 90-day postoperative complications [7].

While a marked decrease in the use of MIS in early-stage cervical cancer following the LACC trial data has been well documented, there remains a gap in understanding the demographics and tumor features of patients still undergoing MIS radical hysterectomy. Furthermore, it is unclear how the absence of a minimally invasive surgical option has affected the selection between surgery versus primary radiation in early-stage cervical cancer and whether the shift in surgical approach has impacted the use of radiation after radical hysterectomy. Utilizing the National Cancer Database (NCDB), our objective was to identify trends in the management of early-stage cervical cancer before and after the publication of the LACC trial concerning MIS versus open surgery and primary surgery versus chemoradiation. We also analyzed clinical and demographic factors associated with the use of MIS in both cohorts and compared the rates of postoperative radiation and outcomes before and after the trial.

## 2. Materials and Methods

### 2.1. Data Source and Patient Selection

Data for this analysis were obtained from the NCDB, a national oncologic registry which includes data from patients who have been treated at Commission on Cancer-accredited centers and covers approximately 70% of newly diagnosed cancer cases in more than 1500 hospitals in the United States [8]. The dataset is de-identified, and this study was considered exempt by the Maimonides Medical Center IRB.

Patients with cervical cancer were identified using International Classification of Disease for Oncology, Third Edition (ICD-O-3), primary site codes C53.0–C53.9. Patients with stage IA2 or IB1 disease were included, based on AJCC clinical and pathologic variables, which correspond with FIGO 2009 cervical cancer staging across all time periods examined. These patients were then categorized as undergoing primary surgery or primary chemoradiation. Those undergoing primary surgery were identified based on site-specific surgical codes 30, 40, 50–54, or 60–62, and patients were excluded if they had undergone neoadjuvant chemotherapy or pre-hysterectomy radiation based on codes for sequence of therapy and surgery. From those patients who did not meet the site-specific surgical codes, patients were excluded if they did not receive radiation or received chemotherapy only based on codes for primary treatment, and the remaining patients in this cohort were identified as receiving primary chemoradiation. We then extracted the following variables: age at diagnosis, race and ethnicity, insurance status, Charlson Comorbidity score, histology, and tumor size. For primary surgery patients, variables for length of postoperative inpatient stay, 30-day readmission and mortality, lymphovascular space invasion (LVSI), margin status, sampled lymph nodes, and adjuvant radiation were extracted.

### 2.2. Statistical Analysis

Once the entire population of early cervical cancer patients who underwent either primary surgery or primary chemoradiation was compiled, the patients were categorized into two subgroups based on the year of diagnosis and its relationship to the year that the LACC trial was published (2018). Those patients diagnosed between 1/2016 and 12/2017 were labeled as the pre-LACC cohort, and the patients diagnosed between 1/2019 and 12/2020 were labeled the post-LACC cohort. Within these two cohorts, an additional comparison was made between those undergoing primary minimally invasive and primary open radical hysterectomy. Using the site-specific surgical codes published in the NCDB Participant User File (2020 Data Dictionary), patients with surgical codes 50–54 were used to specify radicality of hysterectomy, and approach was delineated based on the variable “Approach—Surgery of the Primary Site at this Facility”. Surgeries that were coded as robotic-assisted (code 1) or laparoscopic (code 3) were labeled as minimally invasive, and those that were open (code 5) or converted to open from MIS (codes 2 and 4) were labeled as open.

Descriptive statistics were used to summarize patient cohort characteristics using n (%) for categorical variables and mean (standard deviation) for continuous variables. Wilcoxon’s rank sum or two-sample *t*-test were used to compare continuous characteristics, and Pearson’s chi-squared or Fisher’s exact test were used to compare categorical characteristics between pre-LACC and post-LACC as appropriate. All statistical analyses were performed using R version 4.3.0 (R Foundation for Statistical Computing, Vienna, Austria). All *p*-values are two-sided with statistical significance evaluated at the 0.05 alpha level.

## 3. Results

### 3.1. Demographic, Clinical, and Tumor Characteristics Pre- and Post-LACC

A total of 7930 patients met the inclusion criteria, 4609 of which were diagnosed and received treatment prior to the publication of the LACC trial and 3321 after (Table 1). There were no significant differences between the two cohorts with respect to the age, race, or insurance status. The post-LACC cohort was more likely to have stage IA2 disease (17% vs. 13% prior to trial publication, *p* < 0.001), non-squamous cell histology (37% adenocarcinoma vs. 34% prior to trial publication, *p* = 0.038) and LVSI present in tumors (32% vs. 31%, *p* = 0.008). As the stage of disease was coded based on FIGO 2009 staging, stage IB1 includes all tumors less than or equal to 4 cm. There were no differences in the tumor size between the cohorts, with 46% in each cohort having tumors less than or equal to 2 cm and 34% reporting tumors between 2 and 4 cm. For 20% of the patients in each cohort, the tumor size was unknown based on the available database variables. Staging as recorded in the NCDB during these time periods also did not take into account lymph node positivity, which was similar between the two cohorts, at approximately 20% in each.

After the publication of the LACC trial, the patients were equally as likely to undergo primary hysterectomy but were significantly less likely to undergo minimally invasive hysterectomy (35% vs. 67%, *p* < 0.001). There was no difference in the overall rate of radical hysterectomy before and after the trial (46% vs. 47%, *p* = 0.2), as shown in Table 2. Of those who underwent MIS, 49% had a radical hysterectomy pre-LACC compared to 39% post-LACC (Table S1). Pre-LACC, 85% of the radical MIS cases were robotic-assisted, and post-LACC, 63% of the radical MIS cases were robotic-assisted (Table S2). Of those undergoing an open hysterectomy, 54% had a radical surgery pre-LACC compared to 60% post-LACC (*p* < 0.001) (Table S1).

### 3.2. Postoperative Outcomes and Adjuvant Radiation

For surgical cases, the length of hospital stay was longer in the post-LACC cohort (2.77 vs. 1.98 days, *p* < 0.001). There was no difference in the postoperative mortality rate between the two cohorts (0.2%). The rate of patients receiving postoperative radiation was the same in the pre- and post-LACC cohorts (31% among all the surgical cases, 26% pre- and 24% post-LACC for the radical surgeries only) (Table 3).

### 3.3. Pre- and Post-LACC Comparison of Radical MIS and Open Cases

In the pre-LACC period, patients undergoing radical minimally invasive hysterectomies were more likely to be of White race compared to those undergoing open radical surgery (82% vs. 77%, *p* = 0.001). The patients with private insurance were more likely to undergo MIS compared to open surgery (63% vs. 54%, *p* = 0.004). In the post-LACC period, these race and insurance differences did not persist (Table 4). In both the pre- and post-LACC periods, there were no differences in the mean age between those patients who underwent MIS compared to open radical hysterectomy. There were also no differences in the Charlson Comorbidity Index in either time period.

In the pre-LACC period, there was no difference in the stage of the disease, but patients undergoing radical MIS more frequently had smaller tumors (≤2 cm, 48% MIS vs. 41% open, *p* = 0.023) without a difference in LVSI or positive margins. In the post-LACC period, the patients undergoing radical MIS were more likely to have microscopic disease (stage IA2, 22% vs. 15%, *p* = 0.002). In the 2 years prior to the publication of the LACC trial, the patients undergoing radical open surgery compared to MIS were more likely to receive adjuvant radiation (30% vs. 24%, *p* = 0.011), but there was no difference post-LACC (Table 3). In both time periods, there were significant differences in the lymph nodes being sampled, with a higher frequency sampled among the patients who underwent MIS radical hysterectomy in the pre-LACC cohort (99% vs. 97%, *p* = 0.01) and a higher frequency sampled among the patients who underwent open radical hysterectomy in the post-LACC cohort (99% vs. 96%, *p* < 0.001). In the post-LACC cohort, there was no difference in the positivity of the lymph nodes, whereas there were significantly more patients with positive lymph nodes on final pathology who underwent open as opposed to MIS radical hysterectomy in the pre-LACC cohort (17% open vs. 11% MIS, *p* < 0.001).

### 3.4. Trends in Primary Chemoradiation

When comparing the population who underwent primary hysterectomy to those who received primary chemoradiation, those who underwent chemoradiation were older (mean age 54.82 vs. 47.14, *p* < 0.001), had more comorbidities as measured by the Charlson Comorbidity Index ≥1 (21.7% vs. 14.6%, *p* < 0.001), and more often had public insurance (50% vs. 33%, *p* < 0.001). The patients undergoing primary chemoradiation were less likely to have non-squamous cell histology (19% vs. 37%, *p* < 0.001), and more often had macroscopic disease (92% were classified as stage IB1 vs. 85% of those undergoing primary surgery, *p* < 0.001) (Table S3).

There were no overall differences in the percentage of patients chosen for primary hysterectomy compared to primary chemoradiation, with 8% of the pre-LACC population undergoing primary chemoradiation compared to 7% post-LACC (Table 2). Compared to before the trial’s publication, the post-LACC patients treated with primary chemoradiation were younger (mean age 52 vs. 56, *p* = 0.005) and more likely to have microscopic (stage IA2) disease (13% vs. 5%, *p* = 0.002). The Charlson Comorbidity Index was not significantly different between the two time periods, and there were no differences in insurance or race and ethnicity (Table 5). Patients chosen to undergo primary chemoradiation did not differ between the time periods in terms of the size of the tumor, histology, or presence of LVSI. 

## 4. Discussion

The LACC trial results were first presented in 2018. Prior to this, the use of minimally invasive techniques for radical hysterectomies was rising, especially with the use of robotic-assisted technology [9]. The report of significantly worse overall survival for patients undergoing MIS compared to open surgery resulted in a decline in the use of MIS in early-stage cervical cancer [6]. In this study, we aim to assess if the shift from MIS to open surgery had an impact on the selection of the primary treatment modality (surgery versus radiation) and to identify the demographics and clinical factors influencing the decision to perform MIS in the post-LACC era. Furthermore, we evaluate the impact of the change in surgical approach on the postoperative outcomes, including the need for radiation therapy after radical hysterectomy.

Consistent with previously reported data, our analysis of the NCDB reveals a decrease in the utilization of MIS in early-stage cervical cancer. Before the LACC trial publication, 67% of the patients underwent a minimally invasive hysterectomy, compared to 35% after publication. However, the rate of primary hysterectomy remained stable in the overall population of patients with stage IA2-IB1 cervical cancer, with 92% before and 93% after the publication. Patients referred for primary chemoradiation were generally older and had more comorbidities than those undergoing primary hysterectomy. Among the patients who underwent primary chemoradiation, those that were post-LACC were younger and more likely to have microscopic disease compared to pre-LACC. This suggests that, in the absence of an oncologically safe minimally invasive surgical option, more young patients and those with microscopic disease may be directed to primary chemoradiation.

Since the publication of the LACC trial, debate has ensued regarding the mechanism of the shortened survival associated with and the potential safe utilization of MIS for appropriate candidates [2,4,10]. The choice of surgical modality is also influenced by resource availability and various socioeconomic factors. Discrepancies in the population undergoing MIS versus open surgery before and after the trial underscore surgeons’ preferences and access to specific modalities. Before and after the trial, radical MIS was associated with smaller volume disease, with tumors <2 cm in the pre-LACC cohort, and microscopic disease (stage IA2) in the post-LACC cohort. We investigated other pre-operative factors that could be associated with MIS such as older age and high comorbidity index but found no significant differences between radical MIS and open cases in either cohort. However, the socioeconomic characteristics that differed in the pre-LACC cohort, such as race and insurance status, did not differ in the post-LACC cohort. Before publication, patients undergoing MIS were more likely to be White and have private insurance. Studies have shown that Black race is associated with decreased MIS utilization [11], even with universal health insurance [12]. However, despite lower MIS utilization before knowledge of its detrimental effect on survival, this disparity did not mitigate the decreased survival for Black patients [13]. The more proportionate distribution of race and insurance status in the post-LACC cohort undergoing MIS compared to open surgery underscores the impact of evidence provided by the LACC trial on surgeons and facilities with access to MIS.

Regarding the postoperative outcomes, the length of hospital stay increased by one day in the post-LACC cohort (1.98 vs. 2.77 days, *p* < 0.001). There was no difference in the rates of patients requiring radiation after radical hysterectomy before and after the trial (26% vs. 24%, *p* = 0.3) or in the rates of positive surgical margins (4.8% vs. 4.5%, *p* = 0.6). Consistent with the LACC trial data and other studies published simultaneously, there was no difference in the use of adjuvant radiation between patients undergoing MIS versus open surgery. We also examined adjuvant radiation utilization by surgical modality and found that, in the pre-LACC cohort, patients undergoing radical MIS were less likely to receive adjuvant radiation compared to radical open hysterectomy (24% vs. 30%, *p* = 0.011). In this cohort, patients undergoing MIS had smaller tumors and a lower incidence of positive lymph nodes. 

The strengths of our study include the broad population accessed through the NCDB, which reflects a diverse patient population but also a diverse range of physician practice patterns at various community and academic institutions. Furthermore, this is the first study assessing the impact of the change in surgical approach on the selection of primary treatment modality, necessity for post-radical hysterectomy radiation, and incidence of positive surgical margins. The limitations of our study are largely due to its retrospective design. There were several missing data points, especially regarding postoperative morbidity and mortality variables and tumor size. We did not have access to the depth of stromal invasion to completely assess the Sedlis criteria [14] for postoperative radiation indications, nor did we have access to parametrial involvement to complete the assessment of the Peters criteria [15]. While the NCDB includes 70% of new cancer diagnoses and includes both academic and community settings, this analysis may not be applicable to patients treated in underrepresented settings.

## 5. Conclusions

The rates of primary chemoradiation and radiation after radical hysterectomy have remained stable since the LACC trial. After 2018, patients undergoing primary chemoradiation tended to be younger and have higher rates of microscopic disease, highlighting the necessity for an oncologically safe MIS approach. The use of MIS continues to be associated with small volume disease, both before and after the LACC trial. The socioeconomic disparity in the selection of surgical approach noted prior to the LACC trial is not evident in the post-LACC cohort. With the paradigm shift in surgical approach, we anticipate more mature data to determine if this change in practice has influenced survival outcomes.

## Figures and Tables

**Table 1:** Clinicopathologic characteristics before and after the LACC trial publication.

	Pre-LACC	Post-LACC	*p*-Value
	n = 4609	n = 3321	
Age, mean (SD)	47.91 (13.21)	47.42 (12.94)	0.12
Race			0.081
White	3693 (80%)	2616 (79%)	
Black	473 (10%)	348 (10%)	
Asian	233 (5.1%)	165 (5.0%)	
Other	143 (3.1%)	143 (4.3%)	
Unknown	67 (1.5%)	49 (1.5%)	
Ethnicity			**0.003**
Hispanic	673 (15%)	561 (17%)	
Non-Hispanic	3840 (83%)	2712 (82%)	
Unknown	96 (2.1%)	48 (1.4%)	
Insurance			0.5
Private	2761 (60%)	2013 (61%)	
Medicaid/Medicare	1598 (35%)	1151 (35%)	
Uninsured	207 (4.5%)	133 (4.0%)	
Unknown	43 (0.9%)	24 (0.7%)	
Stage			**<0.001**
IA2	597 (13%)	578 (17%)	
IB1	4012 (87%)	2743 (83%)	
Histology			**0.038**
Squamous cell carcinoma	2472 (54%)	1695 (51%)	
Adenocarcinoma	1581 (34%)	1240 (37%)	
Adenosquamous carcinoma	185 (4.0%)	138 (4.2%)	
Unknown	371 (8.0%)	248 (7.5%)	
Tumor size			0.9
≤2 cm	2100 (46%)	1523 (46%)	
>2 cm–≤4 cm	1568 (34%)	1135 (34%)	
Unknown	941 (20%)	663 (20%)	
Charlson Comorbidity Index			0.3
0	3915 (85%)	2833 (85%)	
1	493 (11%)	367 (11%)	
2	119 (2.6%)	66 (2.0%)	
≥3	82 (1.8%)	55 (1.7%)	
LVSI present	1305 (31%)	988 (32%)	**0.008**
Positive lymph nodes	844 (19.8%)	612 (19.9%)	1

Abbreviations: LACC, Laparoscopic Approach to Cervical Cancer; SD, standard deviation; MIS, minimally invasive surgery; LVSI, lymphovascular space invasion. Results are n (%) unless otherwise reported. Bold *p*-values represent statistically significant results.

**Table 2:** Trends in treatment modalities for patients undergoing primary hysterectomy before and after the LACC trial publication.

	Pre-LACC	Post-LACC	*p*-Value
	n = 4257	n = 3090	
Primary hysterectomy	4257 (92%)	3090 (93%)	0.3
Surgery type			**<0.001**
MIS	2853 (67%)	1091 (35%)	
Open	1052 (25%)	1738 (56%)	
Unknown type	352 (8.3%)	261 (8.4%)	
Hysterectomy type			0.6
Simple	1296 (30%)	933 (30%)	
Radical	1975 (46%)	1467 (47%)	
Other	634 (10%)	429 (14%)	
Unknown	352 (8.3%)	261 (8.4%)	

Abbreviations: LACC, Laparoscopic Approach to Cervical Cancer; MIS, minimally invasive surgery. Results are n (%). Bold *p*-values represent statistically significant results.

**Table 3:** Postoperative outcomes and adjuvant radiation utilization before and after the LACC trial publication.

	Pre-LACC n = 4257	Post-LACC n = 3090	*p*-Value
Positive margin	203 (4.8%)	140 (4.5%)	0.6
Adjuvant radiation	1302 (31%)	950 (31%)	0.9
Adjuvant radiation (radical hysterectomy only)	512 (26%)	359 (24%)	0.3
Length of postoperative admission, mean days (SD)	1.98 (4.52)	2.77 (5.77)	**<0.001**
30-day re-admission	137 (3.2%)	100 (3.2%)	>0.9
30-day mortality	8 (0.2%)	5 (0.2%)	

Abbreviations: LACC, Laparoscopic Approach to Cervical Cancer; SD, standard deviation. Results are n (%) unless otherwise reported. Bold *p*-values represent statistically significant results.

**Table 4:** Comparison of minimally invasive and open approach primary radical hysterectomy patients prior to and after the LACC trial publication.

	MIS n = 1409	Pre-LACC Open n = 566	*p*-Value	MIS n = 423	Post-LACC Open n = 1044	*p*-Value
Age, mean (SD)	46.51 (12.48)	47.56 (12.86)	0.092	46.67 (13.01)	45.91 (12.45)	0.4
Race			**0.001**			0.2
White	1154 (82%)	435 (77%)		318 (75%)	821 (79%)	
Black	111 (7.9%)	77 (14%)		45 (11%)	108 (10%)	
Asian	72 (5.1%)	33 (5.8%)		27 (6.4%)	60 (5.7%)	
Other	45 (3.2%)	16 (2.8%)		28 (6.6%)	39 (3.7%)	
Unknown	27 (1.9%)	5 (0.9%)		5 (1.2%)	16 (1.5%)	
Ethnicity			0.14			0.8
Hispanic	206 (15%)	100 (18%)		82 (19%)	196 (19%)	
Non-Hispanic	1175 (83%)	459 (81%)		336 (79%)	839 (80%)	
Unknown	28 (2.0%)	7 (1.2%)		5 (1.2%)	9 (0.9%)	
Insurance			**0.004**			0.8
Private	883 (63%)	305 (54%)		269 (64%)	635 (61%)	
Medicaid/Medicare	445 (32%)	219 (39%)		133 (31%)	357 (34%)	
Uninsured	66 (4.7%)	33 (5.8%)		18 (4.3%)	43 (4.1%)	
Unknown	15 (1.1%)	9 (1.6%)		3 (0.7%)	9 (0.9%)	
Charlson Comorbidity Index			0.2			0.6
0	1206 (86%)	470 (83%)		368 (87%)	900 (86%)	
1	147 (10%)	64 (11%)		42 (9.9%)	103 (9.9%)	
2	36 (2.6%)	17 (3.0%)		9 (2.1%)	21 (2.0%)	
≥3	20 (1.4%)	15 (2.7%)		4 (0.9%)	20 (1.9%)	
Stage			0.7			**0.002**
IA2	177 (13%)	75 (13%)		93 (22%)	159 (15%)	
IB1	1232 (87%)	491 (87%)		330 (78%)	885 (85%)	
Histology			0.061			0.4
Squamous cell carcinoma	729 (52%)	326 (58%)		211 (50%)	544 (52%)	
Adenocarcinoma	518 (37%)	173 (31%)		172 (41%)	387 (37%)	
Adenosquamous carcinoma	52 (3.7%)	19 (3.4%)		11 (2.6%)	43 (4.1%)	
Unknown	110 (7.8%)	48 (8.5%)		80 (19%)	199 (19%)	
Tumor size			**0.023**			0.6
≤2 cm	671 (48%)	234 (41%)		204 (48%)	477 (46%)	
>2 cm–≤4 cm	474 (34%)	224 (40%)		139 (33%)	368 (35%)	
Unknown	264 (19%)	108 (19%)		80 (19%)	199 (19%)	
Positive margin	64 (4.5%)	20 (3.5%)	0.3	9 (2.1%)	27 (2.6%)	0.6
LVSI present	422 (30%)	183 (32%)	0.4	129 (30%)	336 (32%)	0.8
Lymph nodes sampled	1389 (99%)	548 (97%)	**0.01**	408 (96%)	1035 (99%)	<**0.001**
Lymph nodes positive	150 (11%)	97 (17%)	<**0.001**	56 (13%)	127 (12%)	0.6
Adjuvant radiation	343 (24%)	169 (30%)	**0.011**	106 (25%)	253 (24%)	0.7

Abbreviations: LACC, Laparoscopic Approach to Cervical Cancer; SD, standard deviation; MIS, minimally invasive surgery; LVSI, lymphovascular space invasion. Results are n (%) unless otherwise reported. Bold *p*-values represent statistically significant results.

**Table 5:** Clinicopathologic characteristics of patients who underwent primary chemoradiation before and after the LACC trial publication.

	Pre-LACC	Post-LACC	*p*-Value
	n = 352	n = 231	
Age, mean (SD)	56.37 (16.62)	52.47 (16.72)	**0.005**
Race			0.077
White	273 (78%)	169 (73%)	
Black	57 (16%)	43 (19%)	
Asian	13 (3.7%)	8 (3.5%)	
Other	9 (2.6%)	6 (2.6%)	
Unknown	0	5 (2.2%)	
Ethnicity			0.3
Hispanic	37 (11%)	34 (15%)	
Non-Hispanic	309 (88%)	193 (84%)	
Unknown	6 (1.7%)	4 (1.7%)	
Insurance			0.5
Private	157 (45%)	106 (46%)	
Medicaid/Medicare	180 (51%)	112 (48%)	
Uninsured	13 (3.7%)	13 (5.6%)	
Unknown	2 (0.6%)	0	
Charlson Comorbidity Index			0.4
0	279 (79%)	179 (77%)	
1	36 (10%)	32 (14%)	
2	18 (5.1%)	12 (5.2%)	
≥3	19 (5.4%)	8 (3.5%)	
Stage			**0.002**
IA2	19 (5.4%)	29 (13%)	
IB1	333 (95%)	202 (87%)	
Histology			0.13
Squamous cell carcinoma	254 (72%)	154 (67%)	
Adenocarcinoma	58 (16%)	52 (23%)	
Adenosquamous carcinoma	17 (4.8%)	6 (2.6%)	
Unknown	23 (6.5%)	19 (8.2%)	
Tumor size			0.8
≤2 cm	93 (26%)	55 (24%)	
>2 cm–≤4 cm	136 (39%)	93 (40%)	
Unknown	123 (35%)	83 (36%)	
LVSI present	72 (20%)	47 (20%)	0.2

Abbreviations: LACC, Laparoscopic Approach to Cervical Cancer; SD, standard deviation; LVSI, lymphovascular space invasion. Results are n (%) unless otherwise reported. Bold *p*-values represent statistically significant results.

## Data Availability

The data presented in this study are available from the National Cancer Database.

## Supplementary Materials

The following supporting information can be downloaded at https://www.mdpi.com/article/10.3390/curroncol31050215/s1, Table S1: Trends in radicality of surgery for patients undergoing primary hysterectomy before and after the LACC trial publication; Table S2: Trends in surgical approach for patients undergoing primary radical hysterectomy before and after the LACC trial publication; Table S3: Clinicopathologic characteristics of patients who underwent primary chemoradiation compared to primary surgery.
