# Supplementary material for: Trends in Early-Stage Cervical Cancer Management in the US: A National Cancer Database Analysis
Source: Curr Oncol. 2024 May 16;31(5):2836–45. doi: 10.3390/curroncol31050215 (PMC11119135; doi:10.3390/curroncol31050215)
Supplement: Supplementary file 1 [file curroncol-31-00215-s001.zip › Supplemental Table S1.pdf]

Trends in Early-Stage Cervical Cancer Management in the US: A National Cancer Database Analysis

Supplemental Table S1. Trends in radicality of surgery for patients undergoing primary hysterectomy before and after the LACC trial publication.

	**Pre-LACC**<br>n=4,257	**Post-LACC**<br>n=3,090	**p-value**
MIS Hysterectomy			**<0.001**
Simple	990 (35%)	491 (45%)	
Radical	1409 (49%)	423 (39%)	
Other	454 (16%)	177 (16%)	
Open Hysterectomy			**0.0049**
Simple	306 (29%)	442 (25%)	
Radical	566 (54%)	1044 (60%)	
Other	180 (17%)	252 (14%)	

Abbreviations: LACC, Laparoscopic Approach to Cervical Cancer; MIS, Minimally Invasive Surgery. Results are n (%).
