# Supplementary material for: Trends in Early-Stage Cervical Cancer Management in the US: A National Cancer Database Analysis
Source: Curr Oncol. 2024 May 16;31(5):2836–45. doi: 10.3390/curroncol31050215 (PMC11119135; doi:10.3390/curroncol31050215)
Supplement: Supplementary file 1 [file curroncol-31-00215-s001.zip › Supplemental Table S2.pdf]

Trends in Early-Stage Cervical Cancer Management in the US: A National Cancer Database Analysis

Supplemental Table S2. Trends in surgical approach for patients undergoing primary radical hysterectomy before and after the LACC trial publication.

	**Pre-LACC**<br>MIS, n=2,853<br>Open, n=1,052	**Post-LACC**<br>MIS, n=423<br>Open, n=1044
MIS		
Laparoscopic	208 (15%)	158 (37%)
Robotic-assisted	1201 (85%)	265 (63%)
Open		
Open	537 (95%)	1026 (98%)
Laparoscopic converted to open	11 (1.9%)	12 (1.1%)
Robotic converted to open	18 (3.2%)	6 (0.6%)

Abbreviations: LACC, La0paroscopic Approach to Cervical Cancer; MIS, Minimally Invasive Surgery. Results are n (%).
