# Supplementary material for: Trends in Early-Stage Cervical Cancer Management in the US: A National Cancer Database Analysis
Source: Curr Oncol. 2024 May 16;31(5):2836–45. doi: 10.3390/curroncol31050215 (PMC11119135; doi:10.3390/curroncol31050215)
Supplement: Supplementary file 1 [file curroncol-31-00215-s001.zip › Supplemental Table S3.pdf]

Trends in Early-Stage Cervical Cancer Management in the US: A National Cancer Database Analysis

Supplemental Table S3. Clinicopathologic characteristics of patients who underwent primary chemoradiation compared to primary surgery.

	**Primary Chemoradiation** n=583	**Primary Hysterectomy** n=7,347	**p-value**
Age, mean (SD)	54.82 (16.75)	47.14 (12.59)	**<0.001**
Race			**<0.001**
White	442 (76%)	5867 (80%)	
Black	100 (17%)	721 (9.8%)	
Asian	21 (3.6%)	377 (5.1%)	
Other	15 (2.6%)	271 (3.7%)	
Unknown	5 (0.9%)	111 (1.5%)	
Ethnicity			0.061
Hispanic	71 (12%)	1163 (16%)	
Non-Hispanic	502 (86%)	6050 (82%)	
Unknown	10 (1.7%)	134 (1.8%)	
Insurance			**<0.001**
Private	263 (45%)	4511 (61%)	
Medicaid/Medicare	292 (50%)	2457 (33%)	
Uninsured	26 (4.5%)	314 (4.3%)	
Unknown	2 (0.3%)	65 (0.9%)	
Charlson Comorbidity Index			**<0.001**
0	458 (79%)	6290 (86%)	
1	68 (12%)	792 (11%)	
2	30 (5.1%)	155 (2.1%)	
≥3	27 (4.6%)	110 (1.5%)	
Stage			**<0.001**
IA2	48 (8.2%)	1127 (15%)	
IB1	535 (92%)	6220 (85%)	
Histology			**<0.001**
Squamous cell carcinoma	408 (70%)	3759 (51%)	
Adenocarcinoma	110 (19%)	2711 (37%)	
Adenosquamous carcinoma	23 (3.9%)	300 (4.1%)	
Unknown	42 (7.2%)	577 (7.9%)	
Tumor size			**<0.001**
≤2cm	148 (25%)	3475 (47%)	
>2cm - ≤4cm	229 (39%)	2474 (34%)	
Unknown	206 (35%)	1398 (19%)	
LVSI present	119 (20%)	2293 (31%)	**<0.001**

Abbreviations: LACC, Laparoscopic Approach to Cervical Cancer; SD, standard deviation; LVSI, lymphovascular space invasion. Results are n (%) unless otherwise reported.
